# New adverse coronary events in valve-in-valve TAVR and native TAVR—A 2-year matched cohort

**DOI:** 10.3389/fcvm.2022.1004103

**Published:** 2022-09-21

**Authors:** Ofir Koren, Vivek Patel, Robert Naami, Edmund Naami, Takashi Nagasaka, Alon Shechter, Sharon Shalom Natanzon, Siamak Kohan, Zev Allison, Addee Lerner, Daniel Eugene Cheng, Tarun Chakravarty, Mamoo Nakamura, Wen Cheng, Hasan Jilaihawi, Raj R. Makkar

**Affiliations:** ^1^Cedars-Sinai Medical Center, Smidt Heart Institute, Los Angeles, CA, United States; ^2^Bruce Rappaport Faculty of Medicine, Technion Israel Institute of Technology, Haifa, Israel; ^3^Internal Medicine, University Hospitals Cleveland Medical Center, Case Western Reserve University School of Medicine, Cleveland, OH, United States; ^4^School of Medicine, University of Illinois, Chicago, IL, United States; ^5^The Department of Cardiology, Gunma University Hospital, Gunma, Japan; ^6^Sackler School of Medicine, Tel Aviv University, Tel Aviv, Israel; ^7^Internal Medicine, Kaiser Permanente Medical Center, Los Angeles, CA, United States; ^8^David Geffen School of Medicine, University of California (UCLA), Los Angeles, Los Angeles, CA, United States; ^9^Heart Valve Center, NYU Langone Health, New York, NY, United States

**Keywords:** transcatheter aortic valve replacement, valve-in-valve, coronary artery calcium score, myocardial infarction, coronary catheterization, coronary artery bypass graft (CABG), propensity score matching (PSM), long-term outcome assessment

## Abstract

**Objective:**

To assess the incidence of new adverse coronary events (NACE) following transcatheter aortic valve replacement (TAVR) and valve-in-valve TAVR (ViV-TAVR).

**Background:**

ViV-TAVR is an accepted treatment for degenerative prostheses among patients with high surgical-risk. TAVR studies have suggested an increased risk of coronary artery obstruction and flow stasis causing thrombus formation. Whether contemporary ViV-TAVR is associated with higher rate of coronary events compared to TAVR is unknown.

**Methods:**

We used data from 1,224 TAVR patients between 2016 and 2021. We propensity-matched patients following ViV-TAVR and TAVR by significant predictors to overcome confounders in patients' baseline characteristics and procedural factors.

**Results:**

The matched population included 129 patients in each group. In line with prior reports, there was a higher in-hospital coronary artery obstruction rate with ViV-TAVR (3.1 vs. 1.6%; *p* = 0.23). Despite this, 2-year cumulative NACE rates were similar between groups (4.7 vs. 6.2%, respectively, *p* = 0.79), with no difference between its components: myocardial infarction (MI) (*p* = 0.210), unplanned coronary catheterization (*p* = 0.477), or coronary artery bypass grafting (CABG) (*p* = 0.998). Moreover, hypoattenuated leaflets thickening (HALT) at 30-day CT was observed in nearly a quarter of the patients with no difference between groups (23.9 vs. 23.1%, HR 1.02, 95% CI 0.50–1.28, *p* = 0.872). The progression rate of the coronary artery calcium score (CACS), assessed in a third of patients, was similar between groups (p log-rank = 0.468, 95% CI 0.12–1.24). Low coronary artery height was an unfavorable predictor for in-hospital coronary obstruction and 2-year NACE rate (HR 1.20 and HR 1.25, *p* = 0.001 and *p* < 0.0001, respectively).

**Conclusion:**

At 2-year follow-up, ViV-TAVR was not associated with a higher rate of myocardial infarction, unplanned catheterization, coronary artery bypass grafting, or hypoattenuated leaflet thickening.

## Impact on daily practice

Increasing the awareness of the risk of coronary artery obstruction with ViV-TAVR promotes -appropriate pre-procedural risk assessment and operative success. In our contemporary series of patients, ViV-TAVR is as safe as TAVR in the immediate post-procedural period and over a course of 2 years.

## Introduction

Transcatheter aortic valve replacement (TAVR) has become the most common therapeutic option for severe aortic stenosis regardless of surgical risk (1, 2). With time, bioprosthetic valves may degenerate, leading to stenosis, regurgitation, or both, and require a second intervention, either by a TAVR or redo open-heart surgery (3–6). Valve-in-Valve-TAVR (ViV-TAVR) is an established option in high surgical-risk patients with a degenerative transcatheter or surgical aortic prosthesis and is associated with a lower mortality rate, fewer complications, and shorter length of stay than redo surgical AVR (7–11).

Thrombosis of transcatheter heart valves (THV) has been reported as an incidental finding on 30-day multidetector computed tomography following TAVR with recent evidence indicating higher incidence of stroke or transient ischemic attack (12, 13). The phenomenon was believed to be the consequence of the hemodynamic changes inside the neo-sinus due to the new geometry created by the THV. *In vitro* studies simulating a TAVR environment supported the hypothesis by demonstrating flow stasis within the neo-sinus affecting the coronary flow (14–16). However, a recent multicenter registry showed no clinical correlation between hypoattenuated leaflets thickening (HALT) and combined endpoints of death, stroke, and re-admission due to heart failure after TAVR (17).

When considering the lifetime management of aortic stenosis (AS), several authors have raised concerns on the impact of the implantation of a second (and additional) transcatheter heart valve (18–23) on coronary flow and sinus thrombosis potentially impacting coronary flow.

Our null hypothesis assumed that stasis at the neo-sinuses level resulting from the deployment of a transcatheter valve poses a higher risk for new adverse coronary artery events after the TAVR procedure. Therefore, we aimed to assess the frequency of immediate and later coronary events such as myocardial infarction, unplanned coronary catheterization, coronary artery bypass graft surgery (CABG), and death following ViV-TAVR, in comparison to native TAVR. Moreover, we sought to compare differences in related imaging findings of coronary artery calcium score (CACS) progression and HALT.

## Methods

### Study cohort and patients' selection

We conducted a retrospective study of 1,224 consecutive TAVR patients performed at Cedars-Sinai Medical Center between January 2016 and December 2021 using the new generation intra-annular balloon-expandable Sapien-3 or Sapien-Ultra valve (Edwards Lifesciences, Irvine, California, USA), and the supra-annular self-expanding Evolut-R or Evolut-PRO (Medtronic, Minneapolis, MN, USA). Following CT-TAVR protocol, the consensus decision of the multidisciplinary cardiac team determined the indication for TAVR for all patients, including known anatomical predictors of coronary events (i.e., low coronary height and lengthy leaflets). A shared decision of the interventional team and the attending physicians determined the time of discharge. There were no absolute criteria by which TAVR was declined due to concern for coronary events. In a case of extensive coronary calcification or indetermined coronary calcification on CT-TAVR, diagnostic angiography was performed. In a matter of significant coronary disease seen on CT-TAVR or following diagnostic angiography, percutaneous coronary intervention was completed in a staged fashion. Nearly 20% of patients in each arm of our study underwent complete revascularization within 30 days of their index procedure. Exclusion criteria included the use of experimental THVs, non-transfemoral access sites, incomplete medical records, or if lost to follow-up.

The primary endpoint was a composite of myocardial infarction, unplanned coronary catheterization, percutaneous coronary intervention, and coronary artery bypass surgery up to 2 years following the procedure. Secondary outcomes were the incidence of HALT or the related findings of leaflet hypoattenuation affecting motion (HAM), and reduced leaflet motion (RELM) in a 30-day follow-up CT and the progression of coronary artery disease assessed by the rate of change in coronary artery calcium score.

**Figure 1:**
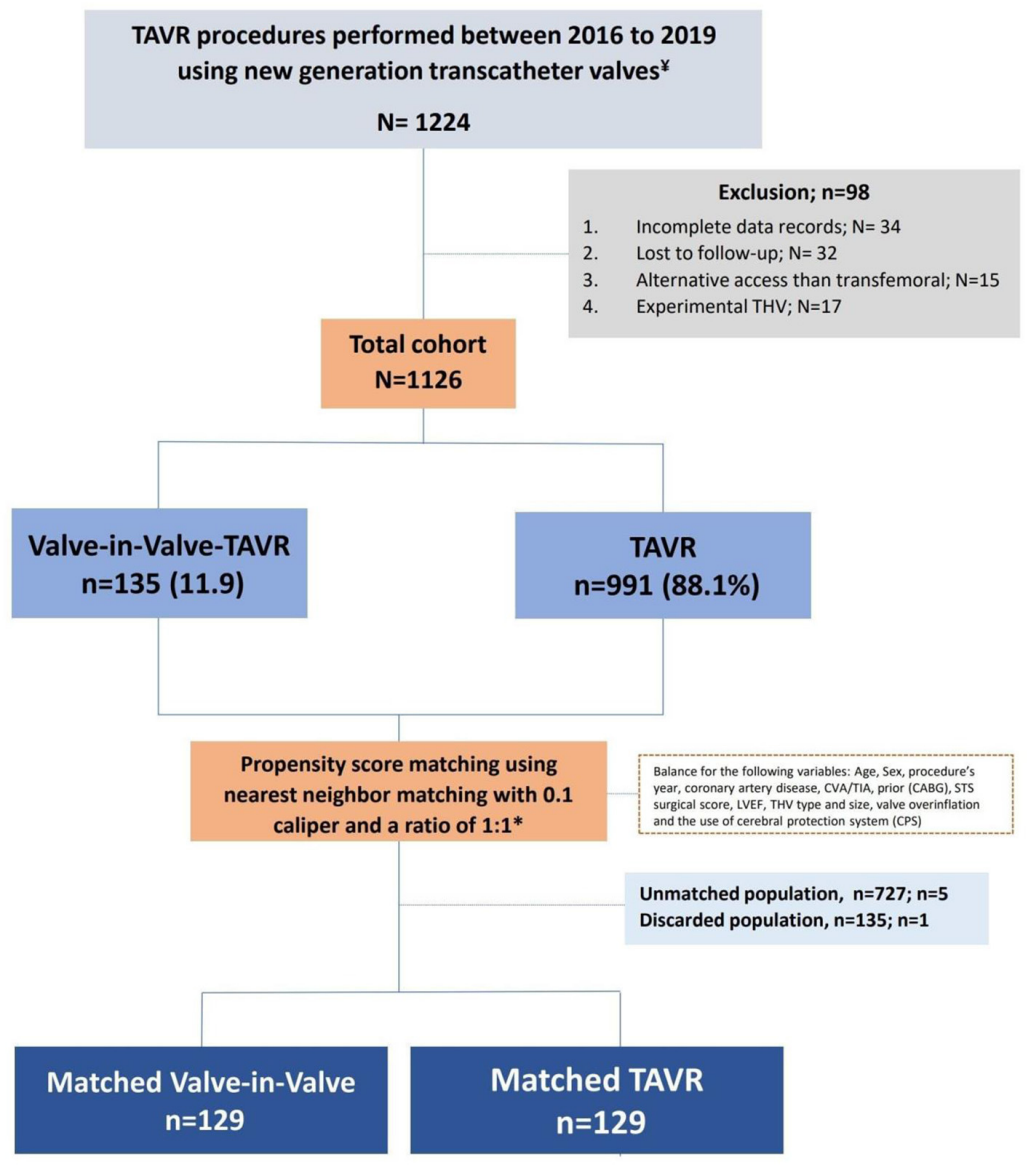
Study design.

We used the data of 1,126 eligible patients for propensity matching and compared the outcome between the two groups (Figure 1). The study was approved by the Cedars-Sinai medical center institutional review board (IRB), which also waived the requirement to obtain informed consent due to the study's retrospective nature.

### Definitions

In-hospital complications such as major bleeding, major vascular complications, and acute kidney failure were followed according to the valve academic research consortium-3 definition of appropriate clinical endpoints in trials involving surgical and transcatheter aortic valves (24).

### Data collection

Demographic, procedural, and follow-up data were entered retrospectively by a dedicated team and extracted using the Cedars-Sinai electronic records systems (CS-link). HALT was assessed using the 2D MPR-diastolic phase (R-R, 80%) to ensure coaptation or presence of HALT and confirmed using the 4D VR-CT-systolic phase (R-R, 20%). The en-face view to assess the extent of RELM. HAM was defined as HALT with RELM of more than 50% as HAM (25). Follow-up CT to assess HALT was randomly assigned between ViV-TAVR and native valve patients.

Coronary artery calcium scores were calculated using the Agatston method using dedicated semi-automatic software (Vitrea Advanced, Vital Images, Minnetonka, MN, USA) (26). Coronary artery calcium scores were measured before and after the procedure during follow-up. The rate of CACS progression or regression was calculated using score difference and the median time elapsed between the tests.

### Statistical analysis

Data are presented as number of patients and percentage (%) for categorical variables and a median (IQR, interquartile range) for continuous variables. Patient characteristics were compared among the study groups using a Kruskal-Wallis test, chi-square test, or Fisher's exact test as appropriate. We use a logistic regression model following a univariate analyses of the patient's baseline characteristics and the 2-year outcomes.

Propensity matching analysis score between ViV-TAVR and the TAVR patients was performed using the nearest neighbor matching technique with one-to-one ratio setting significant (*p* < 0.05) variables and non-significant variables that me be related to the outcome (following parsimonious model) as age, sex, procedure year, diagnosis of coronary artery disease (CAD) and stroke, prior coronary bypass surgery (CABG), Society of Thoracic Surgeons (STS) surgical risk score, LV ejection fraction (LVEF), THV type and size, valve overinflation, and a cerebral protection system (CPS). Patients with deviated threshold scores of >0.10 were considered unmatched (26, 27).

CACS progression rate was constructed using of Kaplan–Meier estimates based on the calcium score before and after the procedure and time elapsed between the tests, and were compared with the use of the log-rank test. Time to NACE was analyzed using the Cox proportional regression methods, where death was considered a relevant competing risk and illustrated by the Kaplan-Meyer survival curve.

All statistical analyses were performed using SAS 9.4 (SAS Institute, Inc., Cary, North Carolina) and R package version 4.0.5 with two-sided tests at a significance level of 0.05.

## Results

### Study population

A total of 1,224 patients underwent TAVR at Cedars-Sinai medical center from 2016 to December 2021. We excluded 34 patients due to incomplete records and 32 patients who were lost to follow-up. We also excluded 15 patients who underwent non-transfemoral TAVR and 17 patients who were involved in a clinical study using an experimental transcatheter valve.

Among the 1,126 eligible patients, 135 patients underwent ViV-TAVR (“ViV-TAVR” group) procedure and 991 TAVR (“TAVR” group) on the native valve. ViV-TAVR on a prior TAVR was performed in 8 (5.9%) patients, while ViV-TAVR on previous surgical AVR was done in 127 (94.1%) patients.

### Baseline characteristics and outcomes among unmatched groups

In an unmatched comparison, the ViV-TAVR patients were significantly older with a median age difference of 2.6 years compared to TAVR patients while comprising similar proportions of females.

There was no difference in the prevalence of common cardiovascular risk factors such as hypertension, hyperlipidemia, diabetes, coronary artery disease, peripheral artery disease, prior myocardial infarction, prior acute coronary syndrome, the use of antiplatelet, or anticoagulation therapy following adjustment for age. The ViV-TAVR group less frequently had a previously placed porcelain aorta and more commonly received prior coronary artery bypass graft surgery (Table 1).

**Table 1:** Baseline clinical characteristics of matched population.

	**All patients (*N* = 258)**	**Valve-in-valve (*N* = 129)**	**TAVR (*N* = 129)**	***p*-value**
Age (years)	79.6 (12)	80.0 (11)	78.8 (13)	0.200
Female sex	116 (45.0)	57 (44.2)	59 (45.7)	0.900
BMI	26.7 (7)	26.8 (8)	26.2 (6)	0.199
Hypertension	146 (56.6)	70 (54.3)	76 (58.9)	0.530
Hyperlipidemia	163 (63.2)	82 (63.6)	81 (62.8)	0.897
Diabetes mellitus	49 (19.0)	23 (17.8)	26 (20.2)	0.751
Porcelain aorta	17 (6.6)	4 (3.1)	13 (10.1)	0.042
Peripheral artery disease	49 (19.0)	26 (20.2)	23 (17.8)	0.751
CKD stage III or higher^ϕ^	35 (13.6)	18 (14.0)	17 (13.2)	1.000
Current dialysis	16 (6.2)	10 (7.8)	6 (4.7)	0.440
Chronic lung disease	50 (19.4)	24 (18.6)	26 (20.2)	0.875
Coronary artery disease	113 (44.0)	55 (43.0)	58 (45.0)	0.802
Myocardial infarction (prior) or ACS	36 (14.0)	18 (14.0)	18 (14.0)	1.000
CABG (prior)	94 (36.4)	46 (35.7)	48 (37.2)	0.897
Left main intervention (prior)	12 (4.7)	5 (3.9)	7 (5.4)	0.769
CVA/TIA (prior)	50 (19.4)	25 (19.4)	25 (19.4)	1.000
Bicuspid aortic valve	4 (1.6)	1 (0.8)	3 (2.3)	0.622
**NYHA class**				
1	1 (0.4)	0 (0)	1 (0.8)	0.066
2	10 (3.9)	7 (5.4)	3 (2.3)	
3	137 (53.1)	76 (58.9)	61 (47.3)	
4	110 (42.6)	46 (35.7)	64 (49.6)	
Coronary artery calcium score, AU	1,119 (2,220)	1,039 (2,409)	1,134 (2,172)	0.906
Aortic valve calcium score, AU	2,340 (3,060)	2,034 (4,655)	2,333 (2,282)	340
STS score	5.6 (5.1)	5.3 (6.5)	5.6 (4.8)	0.437
KCCQ score	52.6 (40)	54.4 (28)	51.5 (40)	0.159
LVEF (%)	59.5 (22)	61.0 (17)	58.5 (25)	0.784
Aortic valve area, cm^2^	0.7 (0.7)	0.7 (0.3)	0.7 (0.8)	0.874
Pre-procedure AV peak gradient, mmHg	60.0 (28)	65.0 (32)	59.5 (27)	0.316
Aspirin use	126 (48.8)	68 (52.7)	58 (45.0)	0.068
PTY12 platelets inhibitors use	55 (21.3)	30 (23.3)	25 (19.4)	0.543
Anticoagulation use	15 (5.8)	8 (6.2)	7 (5.4)	0.726
Diagnostic coronary angiography during TAVR	125 (48.4)	68 (52.7)	57 (44.2)	0.213
Completion of revascularization within 30 days of TAVR	52 (20.2)	30 (23.3)	22 (17.1)	0.277
Use of cerebral protection system (CPS)	152 (58.9)	79 (61.2)	73 (56.6)	0.527
Balloon predilation	6 (2.3)	4 (3.1)	2 (1.6)	0.684
Balloon overinflation^μ^	36 (14.0)	21 (16.3)	15 (11.6)	0.669
Balloon-Expandable THV	215 (83.3)	108 (83.7)	107 (82.9)	1.000
Use of large THV^ψ^	59 (22.9)	30 (23.3)	29 (22.5)	1.000
Fluoroscopy time, min	12.7 (10.7)	16.2 (7.8)	11.3 (9.0)	< 0.0001
Contrast volume, ml	70.0 (58.8)	72.5 (82)	70.0 (50.0)	0.661
Length of stay, days	2.0 (3)	2.0 (5)	2.0 (3)	0.531
In-Hospital complications	11 (4.3)	6 (4.6)	5 (3.9)	0.645
In-hospital coronary obstruction	6 (2.3)	4 (3.1)	2 (1.6)	0.234

TAVR, transcatheter aortic valve replacement; BMI, body mass index; CKD, chronic kidney disease; CABG, coronary artery bypass graft; ACS, acute coronary syndrome; CVA/TIA, cerebrovascular accident/Transient ischemic attack; NYHA, New-York heart association; STS, society of thoracic surgeon; KCCQ, Kansas city cardiomyopathy questionnaire; LVEF, left ventricular ejection fraction; PCI, percutaneous coronary interventions; THV, transcatheter heart valve.

ϕ Large THV referred to 29 mm Sapien valve or 34 mm Evolute valve.

ψ Based on GFR level according to the National Kidney Foundation (NKF).

Data are presented as number of patients (column %) or median (IQR, interquartile range). P-value is calculated by Kruskal-Wallis test for continuous variables, and chi-square or Fisher's exact test for categorical variables as appropriate.

μ For balloon expanding THV only.

Propensity matching was performed for the variables: age, sex, procedure year, coronary artery disease, CVA/TIA, prior coronary artery bypass graft surgery (CABG), STS surgical score, LVEF, THV type and size, balloon overinflation and the use of cerebral protection system (CPS) using the nearest neighbor matching with 0.1 caliper and a ratio of 1:1. Overall balance test (28) p = 0.957.

The ViV-TAVR patients had an overall higher STS score (7.1 vs. 5.4), lower median LVEF, and were more likely to receive a CPS.

The use of an intra-annular balloon-expandable THV and a large-size THV with post-procedural overinflation was more commonly reported in the ViV-TAVR group (Table 1).

With regards to in-hospital adverse events, including major bleeding, vascular complications, renal failure, stroke, coronary obstruction, conversion to open surgery, and death, there was no difference between the two groups. When comparing the unmatched cohorts, the rate of coronary artery obstruction in ViV-TAVR was higher than reported in the TAVR group, but this difference was not statistically significant (Table 2).

**Table 2:** Clinical end points at 2 years.

**End point**	**At 2 years**				***p-*value**
	**Entire cohort (*N* = 258)**	**Valve-in-valve (*N* = 129)**	**TAVR (*N* = 129)**	**Hazard ratio (95% CI)**	
**No. of patients (%)**					
Myocardial infarction	5 (1.9)	2 (1.2)	3 (2.3)	0.24 (0.02–2.21)	0.210
Unplanned coronary catheterization	8 (3.1)	3 (2.3)	5 (3.9)	0.59 (0.13–2.52)	0.477
Coronary artery bypass graft surgery	1 (0.4)	1 (0.8)	0 (0)	NA	0.998
NACE	14 (5.4)	6 (4.7)	8 (6.2)	0.94 (0.47–1.86)	0.785
Death	25 (9.7)	13 (10.1)	12 (9.3)	1.00 (0.43–2.31)	0.975

NACE, new adverse coronary event. Composite events of myocardial infarction, unplanned coronary catheterization, and coronary artery bypass graft surgery.

Data are presented as number of patients (column %) or median (IQR, interquartile range).

### Post-procedure medical therapy

The use of dual antiplatelet therapy or, when indicated, an oral anticoagulant (OAC) and single antiplatelet (SAP) following the procedure was similar between the two groups. In the ViV-TAVR group, 86 (63.7%) patients used DAPT and 48 (35.6%) used OAC and SAP, compared to the TAVR group, where 562 (56.6%) patients used DAPT, and 426 (42.9%) used OAC and SAP.

### Two-year outcomes in matched groups

**Figure 2:**
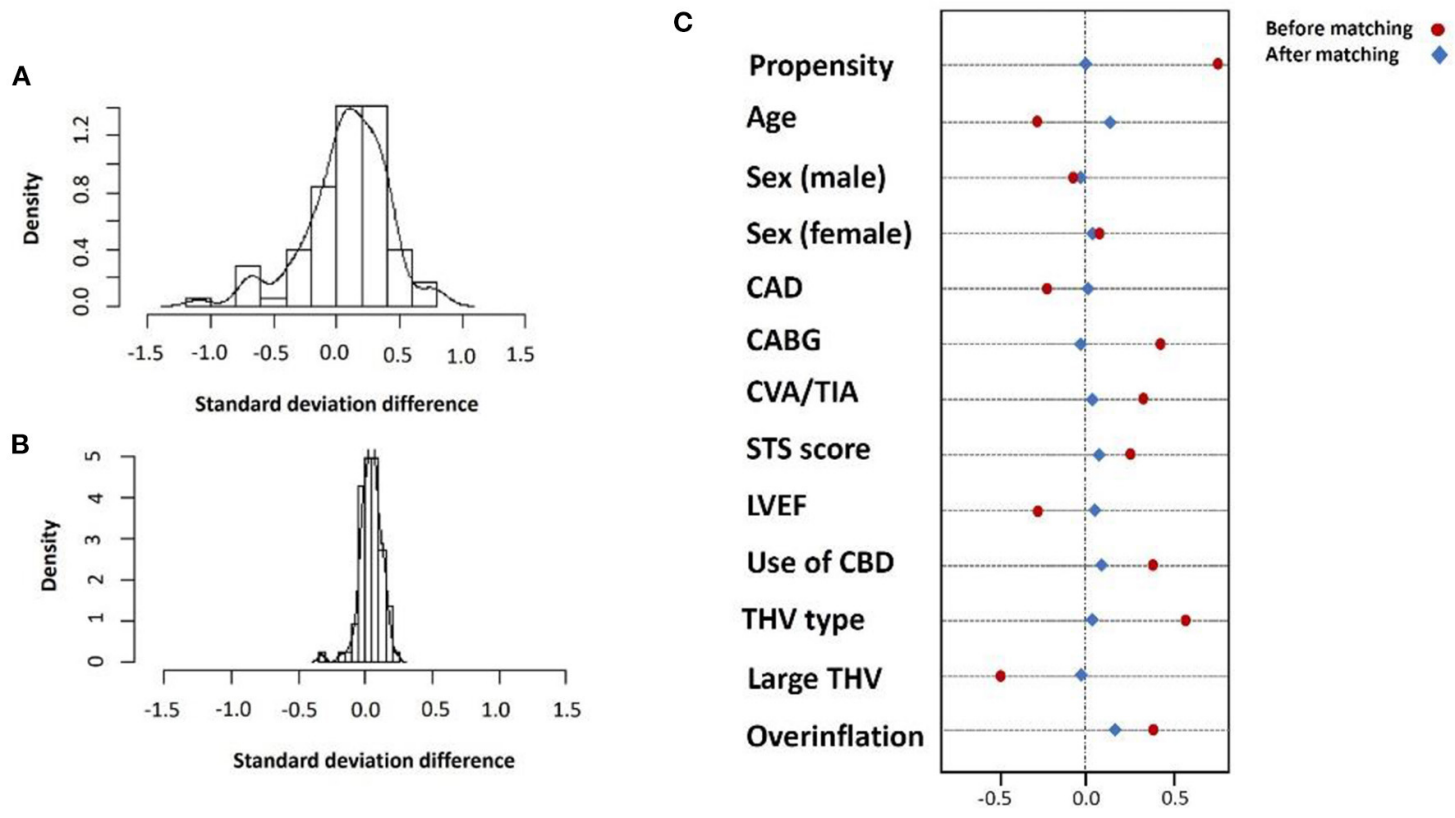
Standard deviation before **(A)** and after **(B)** propensity matching and covariate balance for selected variables **(C)**.

Following the PSM model with a 1:1 ratio, we matched 129 patients in each group to address possible confounders derived from patients' baseline characteristics and procedural-related events by balancing the following covariates: age, sex, year of procedure, the existence of CAD, prior stroke or CABG, surgical score and LVEF, THV type and size, the use of valve overinflation and CPS use (Figure 2).

In the matched population, ViV-TAVR patients had a lower rate of previously placed porcelain aorta (3.1 vs. 10.1%, *p* = 0.042) and greater fluoroscopy time (11.3 vs. 16.2 min, *p* < 0.0001). In both groups, diagnostics coronary angiography was performed in almost half the patients before the procedure, and the use of aspirin, P2Y12 inhibitors or anticoagulation was used at similar rates. Post-procedural guideline-directed pharmaceutical treatment was endorsed in over 95% of patients in both groups. There was no significant difference in overall in-hospital complications, even though the ViV-TAVR group had a higher rate of coronary obstruction (Table 2).

**Figure 3:**
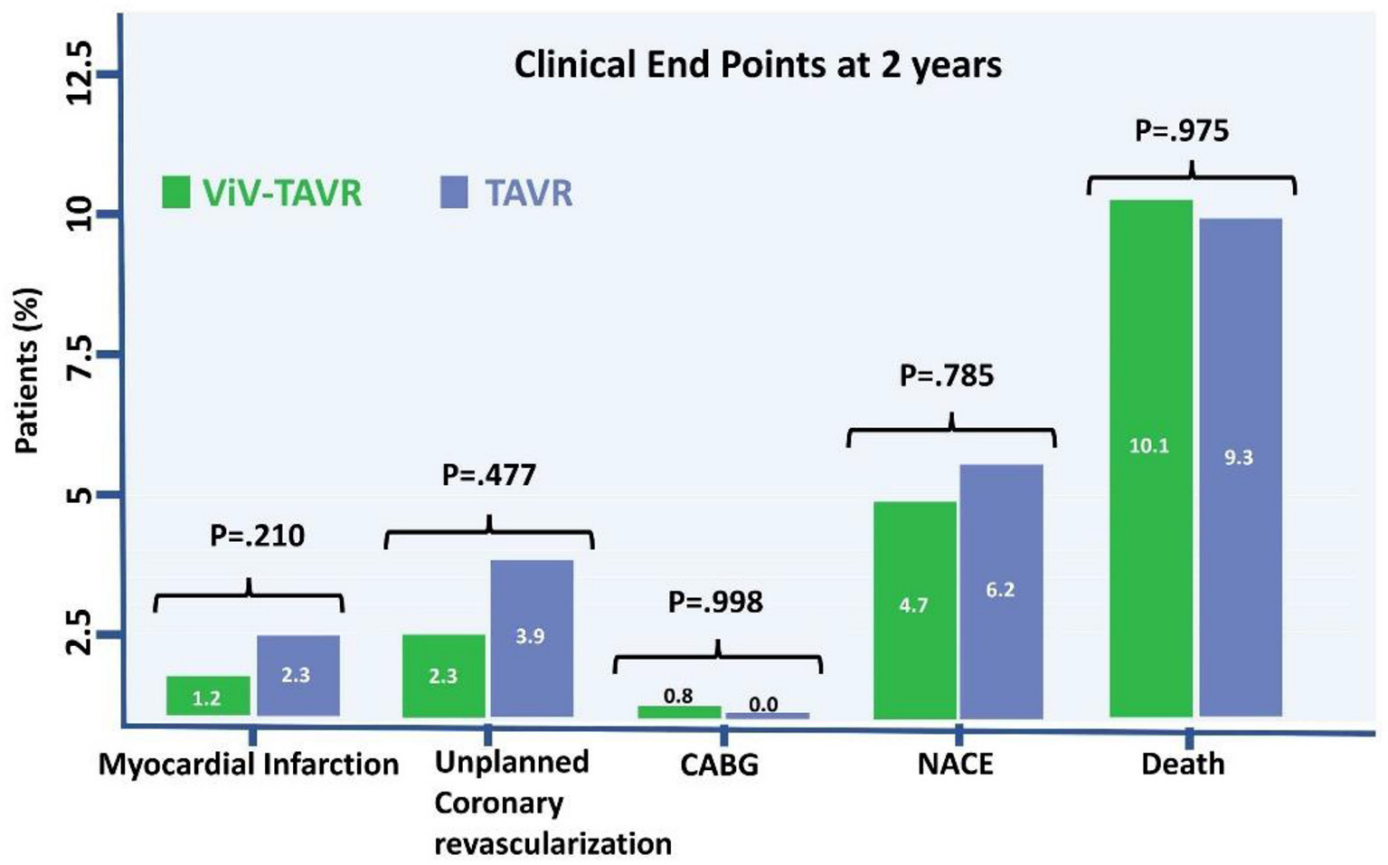
NACE, death, and stroke to 2 years.

**Figure 4:**
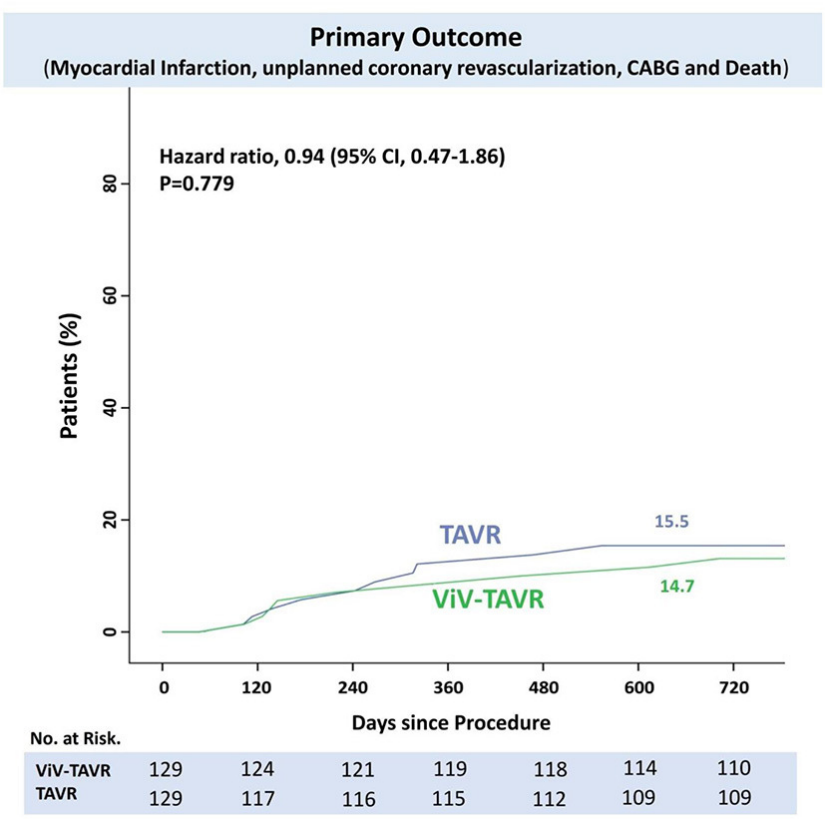
Time-to-events curve of NACE and death to 2 years.

The rate of myocardial infarction, unplanned coronary catheterization, or coronary artery bypass graft composing the primary endpoints during 2-year follow-up was reported in 14 (5.4%) patients with no difference between the groups (Figure 3). Thromboembolic cause of MI was seen in only one patient of the native-TAVR group, while most MI cases presented predominantly as plaque rupture. A total of 25 (9.7%) patients died during follow-up, and the time to the first event composing the primary outcome and 2-year death did not differ between the two groups (Figure 4).

### Hypoattenuated leaflet thickening and reduced leaflet motion

HALT was observed in 23.5% of the 98 patients who had a 30-day follow-up CT. Among the HALT patients, RELM of at least 50% was seen in 4 patients. There was no difference in the HALT and RELM rates between ViV-TAVR and TAVR groups (Table 3).

**Table 3:** CT-based valvular characteristics at 30-day follow-up.

**End point**	**At 30-day^β^**				***p-*value**
	**Total patients *N* = 98**	**Valve-in-valve (*N* = 46)**	**TAVR (*N* = 52)**	**Hazard ratio (95% CI)**	
**No. of patients (%)**					
Hypoattenuated leaflet thickening (HALT)^*^	23 (23.5)	11 (23.9)	12 (23.1)	1.02 (0.50–1.28)	0.872
Hypoattenuation affecting motion (HAM)^ψ^	16 (16.3)/(69.6)^Ω^	7 (15.2)/(63.7)^Ω^	9 (17.3)/(75.0)^Ω^	0.87 (0.38–1.60)	0.776
Reduced leaflet motion (RELM) of < 50%^¥^	4 (4.1)/(17.4) ^Ω^	2 (4.3)/(18.2) ^Ω^	2 (3.8)/(16.7) ^Ω^	1.32 (0.36–4.06)	0.758

* Hypoattenuated leaflet thickening (HALT) referred to a valvular characteristic of Hypoattenuated opacity at the base of vale leaflets on a 4-dimentional volume-rendering CT scan (24).

ψ Hypoattenuated affecting motion (HAM) defined as reduction of leaflets motion in the presence of HALT.

¥ Reduced leaflets motion (RELM) quantified in a systolic phase with a VR en face projection at maximal leaflet opening (24).

Ω In-respect to HALT.

β Median follow-up of 32 days. 30-day follow-up CT was performed in 98 (37.9%) patients.

### Progression of coronary calcification

**Figure 5:**
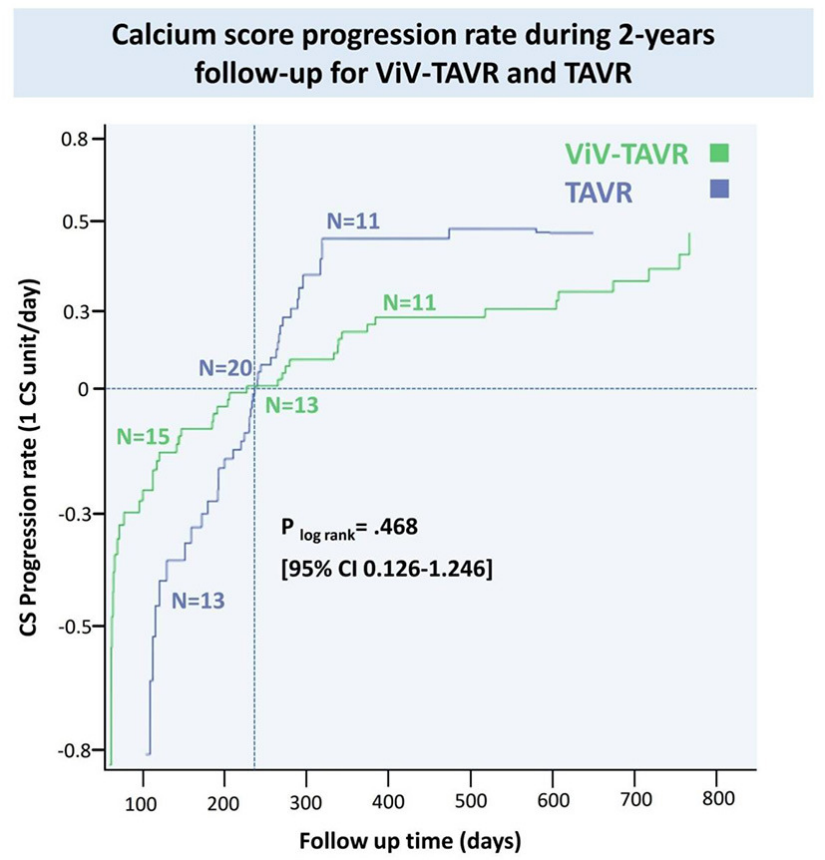
Coronary artery progression rate during 2-years follow-up for ViV-TAVR and TAVR.

Coronary artery calcium score (CACS) using reconstructed CT images before and after the procedure was performed in 82 (31.8%) of the matched population in a median of 605 days. CACS progressed in 22 (8.5%) patients, regressed in 28 (10.8%) patients and did not change in 32 (12.4%) patients. There was no statistical difference in the progression rate of the two groups (Figure 5).

### Anatomical predictors of coronary obstruction and nace

**Figure 6:**
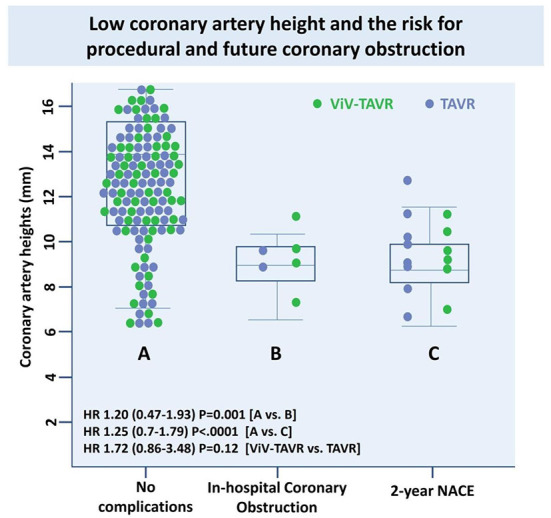
Low coronary artery height and the risk for procedural and future coronary obstruction.

Low coronary height, defined by the distance of either the right or left coronary artery from the coronary ostium to the aortic valve annulus of < 10 mm, was measured in 10 (71.4%) of the NACE patients with no significant difference between ViV-TAVR and TAVR (Table 4). Low coronary height was associated with both in-hospital coronary obstruction and 2-year NACE and did not differ between the groups (Figure 6).

**Table 4:** Anatomical and clinical features of coronary event cases.

**NACE case**	**Study group**	**THV type**	**THV size**	**Annulus area (mm^2^)**	**LM height (mm)**	**RCA height (mm)**	**VTC (L) (mm)**	**VTC (R) (mm)**	SOV (L)^*^(mm)	SOV (R)^*^(mm)	**SOV area (mm^2^)**	**STJ height (mm)**	**VT-STJ (mm)**	**Type of previous SAV**	**HALT**	**CPS**	**In-hospital coronary obstruction**	**All-cause death**	**Time-to-death (days)**
1	TAVR	E-R	22.5	378	8.7	11.2	4.6	5.3	21.5	19.1	411	19.7	1.2	-	-	1	1	0	
2	TAVR	S3	22.5	345	11.8	9.4	3.8	4.6	19.4	17.9	368	17.4	−1.1	-	-	0	1	0	
3	TAVR	S3	22.5	346	8.4	10.5	4.4	4.5	14.8	14.2	404	23.1	4.6	-	+	0	0	1	36
4	TAVR	S3	22.5	565	11.6	7.9	3.8	3.8	25.1	29.4	738	21.4	2.9	-	+	0	0	0	
5	TAVR	S3	18.0	784	11.4	9.8	3.7	2.9	35.5	32.3	894	24.4	10.4	-	-	0	0	0	
6	TAVR	S3	18.0	664	6.8	16.5	4.1	3.9	27.8	26.5	737	16.4	2.4	-	NA	0	0	0	
7	TAVR	S3	20.0	529	11.4	12.4	4.4	4.8	23.3	28.2	657	17.9	1.9	-	NA	0	0	0	
8	TAVR	S3	22.5	380	12.8	11.2	5.2	4.1	17.0	20.4	397	17.4	−1.1	-	-	0	0	0	
9	ViV-TAVR	S3	20.0	431	8.7	6.3	2.3	3.8	22.6	25.3	572	17.2	1.2	Mitroflow	NA	1	1	1	In-hospital
10	ViV-TAVR	E-R	20.0	422	8.8	13.5	4.3	3.3	23.2	24.3	564	22.4	6.4	Magna	+	1	1	1	48
11	ViV-TAVR	S3	20.0	398	11.8	9.8	4.1	4.8	23.7	18.8	446	23.4	7.4	Transcatheter	-	0	0	0	
12	ViV-TAVR	S3	22.5	548	11.4	8.9	2.1	4.4	22.0	28.8	634	26.7	8.2	Mitroflow	+	0	0	0	
13	ViV-TAVR	S3	22.5	588	13.6	10.9	3.8	5.6	24.7	26.0	642	25.8	7.3	Mitroflow	-	0	0	0	
14	ViV-TAVR	S3	20.0	505	11.2	14.6	5.0	4.9	23.5	25.3	595	22.8	6.8	Trifecta	NA	0	0	0	

THV, Transcatheter heart valve; LM, left main; RCA, right coronary artery; VTC, valve to coronary ostium; SOV, sinus of valsalva; STJ, sinotubular junction; VT-STJ, valve to STJ; HALT, hypoattenuated leaflet thickening; CPS, cerebral protection system.

* The distance from the commissure to the sinus.

## Discussion

The ViV-TAVR procedure has emerged as a safe and effective treatment for a degenerated bioprosthetic aortic valve and offers an additional therapeutic option for selected patients over redo surgery (29). Despite the overall high procedural success rate of ViV-TAVR, as well as vigilant CT-guided procedural planning, abrupt coronary obstruction remains a common and devastating complication, with the rate of incidence estimated at 2.5–3.5% (30, 31). Limited data exists on adverse coronary events after discharge.

### Coronary events following TAVR and ViV-TAVR

The prevalence of CAD in TAVR patients is approximately 50%, with approximately half of them undergoing PCI before the procedure. In a cohort of ~800 TAVR patients, Vilalta et al. reported an incidence of ACS in 10% of TAVR patients in a median follow-up time of 2 years with unstable angina and non-ST elevation myocardial infarction constituting the majority of cases (31). Mentias et al. reported similar findings among 140,000 TAVR patients, where 5% were admitted for ACS within 1 year of the procedure and 30% required invasive management (32). The strongest predictors of ACS in the larger cohort included a history of CAD, recent revascularization, and ViV-TAVR (13, 14).

Non-immediate ACS following TAVR is believed to be the consequence of one of two distinct processes: the formation of *de novo* coronary artery plaque and prosthetic valve thrombosis migrating to the coronary vessels. While distinct, both processes are thought to be sequelae from impaired perfusion or flow to the coronary sinuses (17–21). The shorter or more compact sinus of valsalva, following valve deployment, promotes flow stagnation and subsequent platelet aggregation leading to atherothrombosis in the coronary vessel or bioprosthetic valve (22, 23, 33).

Our study assesses the incidence of coronary events such as acute myocardial infarction, unplanned coronary catheterization, and CABG following matched population of ViV-TAVR and TAVR patients. We further assessed the rates of HALT, HAM, RELM, and the progression of coronary calcification.

We report that the cumulative clinical incidence of new adverse coronary events following ViV-TAVR was not statistically different from TAVR patients. ViV-TAVR patients had similar rates of HALT, HAM and RELM as well as and the coronary artery calcium progression. Albeit not significant, complications occurred most commonly in the ViV-TAVR cohort in the setting of previously used externally mounted surgical valves. However, due to the limited number of event rates (i.e., < 5%), it would be difficult to a reliable conclusion. Furthermore, our results indicate that a low coronary height (< 10 mm from the annulus), believed to be an unfavorable predictor for abrupt coronary obstruction during the procedure and future NACE event (34).

A possible explanation for our results is the relatively short follow-up period compared to previous studies assessing TAVR-related outcomes. Makkar et al. reported no differences in death outcomes or disabling stroke in 5 years when comparing TAVR to SAVR among intermediate surgical-risk patients with severe, symptomatic aortic stenosis (35). A sensitivity analysis with various follow-up times may demonstrate appreciable differences in the two cohorts. In our cohort, most of the TAVR and ViV-TAVR patients underwent routine pre-procedural CT with coronary assessment. In half of the borderline cases, a left heart catheterization was performed prior to valve deployment, allowing for the intervention of vulnerable plaque, making subsequent coronary events less likely. Faroux et al. report that nearly 30% of ACS post-TAVR involved untreated lesions present before TAVR (36, 37). In valve technology, newer generation valves are engineered to minimize the risk of common complications albeit technologic needs remain unmet when minimizing risk for periprocedural coronary events. Additionally, more recent clinical data better direct clinicians on appropriate anti-platelet and anticoagulant regimens (38). Ultimately, the work-up, management, and protocol of TAVR have evolved, allowing for more favorable outcomes seen in the ViV cohort. We matched the population to the procedural year and excluded old generation sheaths and devices to address time-related potential confounders.

Another important determinant of coronary events following TAVR is the completeness of revascularization pre-operatively. To date, several earlier studies suggest no association between CAD severity and TAVR outcomes (39–42). These studies however were largely limited in their design in terms of sample size, use of standardized indices for CAD severity (i.e., syntax score), appropriate follow-up time, and use of strictly trans-apically placed valves. Witberg et al., however, reported a strong relationship between severe syntax scores and mortality post-TAVR. While shown previously in single-center observational studies, this was the first report *via* a multicenter analysis and sufficient sample size. Moreover, it reported a powerful relationship with incomplete levels of revascularization which they reported as a syntax score of >8 following the index revascularization (43–46). In our study, ~20% of each cohort underwent full revascularization within 30 days of their TAVR procedure. This may help explain the number of reported coronary complications in each of the studied groups. However, given there was no different in the rate of revascularization, we do not expect this to be a confounding variable in the overall study.

To the best of our knowledge, this is the first study to comprehensively assess clinical coronary adverse events, leaflets thrombosis, and coronary artery calcium score progression in a 2 year period following ViV-TAVR.

### Study limitation

The current study represents the first reported analysis concerning the incidence and outcomes of coronary events following ViV-TAVR in a matched population of TAVR patients. Our investigation has several limitations. First, our cohort is based on single-center data and lacked comprehensive input from a multicenter experience. Second, we equated coronary events to myocardial infarction, unplanned coronary catheterization, and CABG. A more robust characterization would have included readmission due to unstable angina and whether the MI occurred in type 1 or type 2 injury patterns. Third, since our ViV-TAVR cohort enclosed only a small number of patients who had prior transcatheter valves, we could not make a strong assumption about the long-term outcome in those patients. Fourth, our study only followed patients for 2 years in terms of long-term outcomes, which raises questions about whether the differences in outcomes between TAVR and ViV would be more apparent over a more extended follow-up period. Fifth, residual confounding cannot be entirely excluded despite propensity score matching analysis to adjust for measured confounders. Sixth, it is important to note that among our cohort with known CAD, the majority of patient received CABG rather than PCI for revascularization. The concern is that patients undergoing a PCI-based approach for revascularization are at higher risk of adverse outcomes following TAVR. Specifically, post-PCI patients require DAPT therapy which may need to be continued following TAVR placing them at higher risk of bleeding. Additionally, PCI poses a theoretical risk of in-stent thrombosis, particularly with curtailed duration of DAPT. This question was recently addressed by the ACTIVATION trial which compared the relationship between PCI vs. medical therapy in pre-operative TAVR patient with at least one coronary lesion of a major epicardial vessel. Although no difference was found, the study was not carried out to completion due to low enrollment rates. Nevertheless, our findings may have differed if PCI-based revascularization was more prevalent in our studied cohort (47–49). Seventh, several recent studies have demonstrated the role which valve orientation plays in determining risk of acute coronary obstruction and sinus sequestration, particular with supra-annular valve implantation. This information was not evident in procedural summaries of our patients, a potential limiting factor in our study. We therefore could expect poorer outcomes and higher rates of NACE if an operator was not aware of this technically important step in TAVR implantation (34, 50–52).

Lastly, our medical records are exposed to patients hospitalized only at Cedars networks and therefore may underestimate the event rate. However, patients who were lost follow-up or had inadequate medical records were excluded from the study.

## Conclusions

**Figure 7:**
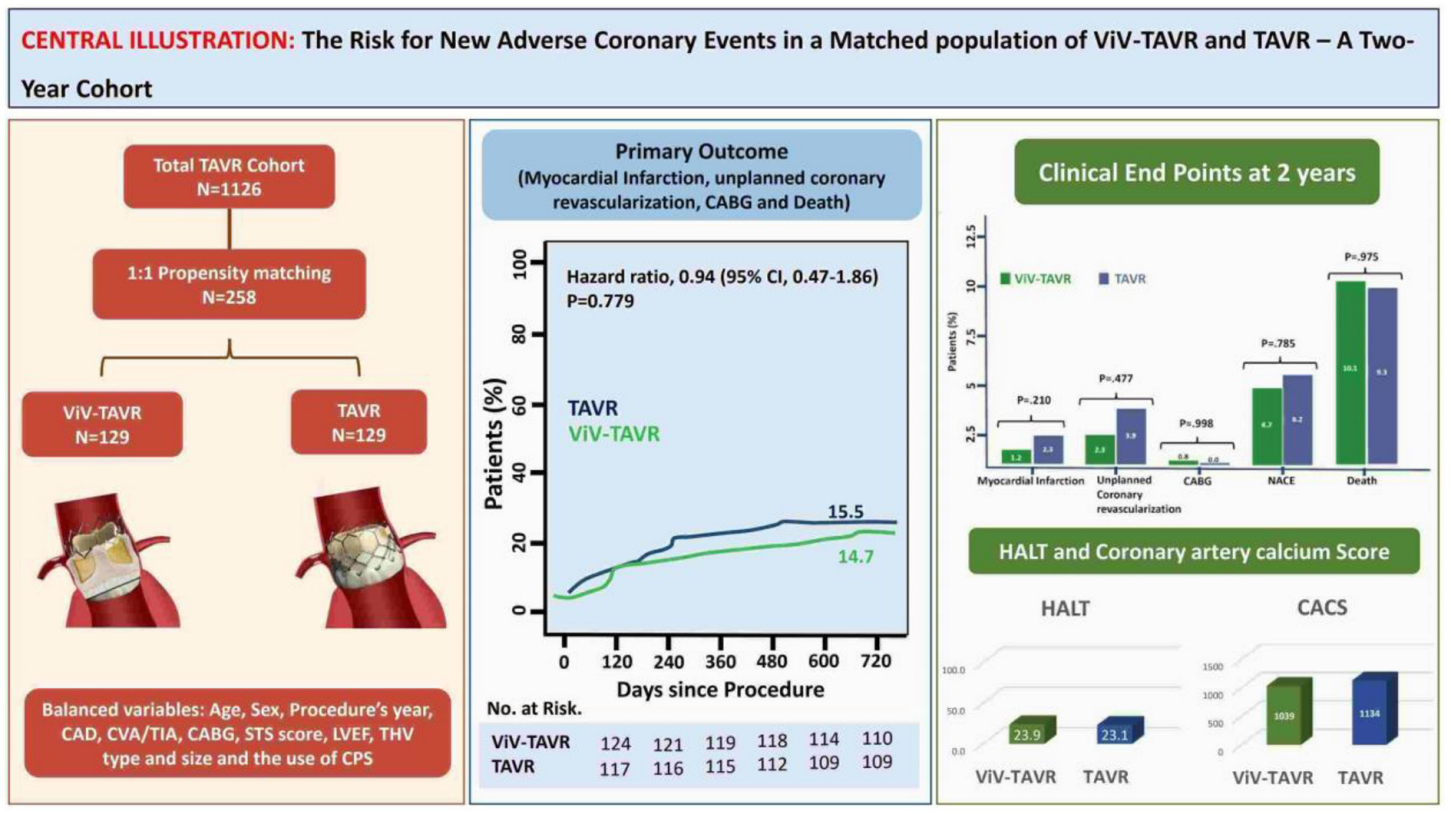
Central illustration.

Our study shows that ViV-TAVR is not associated with a higher risk of coronary events up to 2 years following the procedure. Furthermore, proposed mechanism for increased risk of coronary events, including HALT and RELM, were similar between the groups. Lastly, the acceleration of atherosclerosis, assessed by the coronary artery calcium score was similar between the groups. Follow-up studies are required to determine more long-term outcomes, particularly coronary events, in ViV-TAVR vs. TAVR (Figure 7).

## Data availability statement

The raw data supporting the conclusions of this article will be made available by the authors, without undue reservation.

## Author contributions

OK, VP, HJ, and RM conceptual the hypothesis, validated the results, and supervised all processes of the manuscript. EN, RN, TN, AS, SN, SK, ZA, AL, and DC assist in data collection, statistical analysis, and the final writing. TC, MK, and WC provided a detailed review and helped in the last draft. All authors contributed to the article and approved the submitted version.

## Funding

This study was supported in part by the California Chapter of the American College of Cardiology through the Save a Heart Foundation.

## Conflict of interest

Author RM received grant support from Edwards Lifesciences Corporation; he is a consultant for Abbott Vascular, Cordis, and Medtronic and holds equity in Entourage Medical. Author TC is a consultant, proctor, and speaker for Edwards Lifesciences and Medtronic; he is a consultant for Abbott Lifesciences and a consultant and speaker for Boston Scientific. The remaining authors declare that the research was conducted in the absence of any commercial or financial relationships that could be construed as a potential conflict of interest.

## Publisher's note

All claims expressed in this article are solely those of the authors and do not necessarily represent those of their affiliated organizations, or those of the publisher, the editors and the reviewers. Any product that may be evaluated in this article, or claim that may be made by its manufacturer, is not guaranteed or endorsed by the publisher.

## Abbreviations

AS: Aortic Stenosis
CABG: Coronary artery bypass graft
CAD: Coronary artery disease
CACS: Coronary artery calcium score
HALT: Hypoattenuated leaflets thickening
HAM: Hypoattenuated affecting motion
LVEF: Left ventricle ejection fraction
NACE: New adverse coronary event
PSM: Propensity score matching
RELM: Reduced leaflet motion
TAVR: Transcatheter aortic valve replacement
THV: Transcatheter heart valve
ViV-TAVR: Valve-in-Valve TAVR procedure.
